# A Large-Scale Genome-wide Association Study of Blood Pressure Accounting for Gene-Depressive Symptomatology Interactions in 564,680 Individuals from Diverse Populations

**DOI:** 10.21203/rs.3.rs-6025759/v1

**Published:** 2025-02-17

**Authors:** Songmi Lee, Clint L Miller, Amy R Bentley, Michael R Brown, Pavithra Nagarajan, Raymond Noordam, John Morrison, Karen Schwander, Kenneth Westerman, Minjung Kho, Aldi T Kraja, Paul S de Vries, Farah Ammous, Hughes Aschard, Traci M Bartz, Anh Do, Charles T Dupont, Mary F Feitosa, Valborg Gudmundsdottir, Xiuqing Guo, Sarah E Harris, Keiko Hikino, Zhijie Huang, Christophe Lefevre, Leo-Pekka Lyytikäinen, Yuri Milaneschi, Giuseppe Giovanni Nardone, Aurora Santin, Helena Schmidt, Botong Shen, Tamar Sofer, Quan Sun, Ye An Tan, Jingxian Tang, Sébastien Thériault, Peter J van der Most, Erin B Ware, Stefan Weiss, Wang Ya Xing, Chenglong Yu, Wei Zhao, Md Abu Yusuf Ansari, Pramod Anugu, John R Attia, Lydia A Bazzano, Joshua C Bis, Max Breyer, Brian Cade, Guanjie Chen, Stacey Collins, Janie Corley, Gail Davies, Marcus Dörr, Jiawen Du, Todd L Edwards, Tariq Faquih, Jessica D Faul, Alison E Fohner, Amanda M Fretts, Srushti Gangireddy, Adam Gepner, MariaElisa Graff, Edith Hofer, Georg Homuth, Michelle M Hood, Xu Jie, Mika Kähönen, Sharon LR Kardia, Carrie A Karvonen-Gutierrez, Lenore J Launer, Daniel Levy, Maitreiyi Maheshwari, Lisa W Martin, Koichi Matsuda, John J McNeil, Ilja M Nolte, Tomo Okochi, Laura M Raffield, Olli T Raitakari, Lorenz Risch, Martin Risch, Ana Diez Roux, Edward A Ruiz-Narvaez, Tom C Russ, Takeo Saito, Pamela J Schreiner, Rodney J Scott, James Shikany, Jennifer A Smith, Harold Snieder, Beatrice Spedicati, E Shyong Tai, Adele M Taylor, Kent D Taylor, Paola Tesolin, Rob M van Dam, Rujia Wang, Wei Wenbin, Tian Xie, Jie Yao, Kristin L Young, Ruiyuan Zhang, Alan B Zonderman, The Biobank Japan Project, Lifelines Cohort Study, Maria Pina Concas, David Conen, Simon R Cox, Michele K Evans, Ervin R Fox, Lisa de las Fuentes, Ayush Giri, Giorgia Girotto, Hans J Grabe, Charles Gu, Vilmundur Gudnason, Sioban D Harlow, Elizabeth Holliday, Jonas B Jost, Paul Lacaze, Seunggeun Lee, Terho Lehtimäki, Changwei Li, Ching-Ti Liu, Alanna C Morrison, Kari E North, Brenda WJH Penninx, Patricia A Peyser, Michael M Province, Bruce M Psaty, Susan Redline, Frits R Rosendaal, Charles N Rotimi, Jerome I Rotter, Reinhold Schmidt, Xueling Sim, Chikashi Terao, David R Weir, Xiaofeng Zhu, Nora Franceschini, Jeffrey R O’Connell, Cashell E Jaquish, Heming Wang, Alisa Manning, Patricia B Munroe, Dabeeru C Rao, Han Chen, W James Gauderman, Laura Bierut, Thomas W Winkler, Myriam Fornage

**Affiliations:** Brown Foundation Institute of Molecular Medicine, The University of Texas Health Science Center at Houston, McGovern Medical School, Houston, TX; Center for Public Health Genomics, Department of Public Health Sciences, University of Virginia, Charlottesville, VA; Center for Research on Genomics and Global Health, National Human Genome Research Institute, National Institutes of Health, Bethesda, MD; Human Genetics Center, Department of Epidemiology, The University of Texas Health Science Center at Houston School of Public Health, Houston, TX; Division of Sleep and Circadian Disorders, Department of Medicine, Brigham and Women’s Hospital, Boston, MA; Department of Internal Medicine, Section of Gerontology and Geriatrics, Leiden University Medical Center, Leiden; Division of Biostatistics, Department of Population and Public Health Sciences, University of Southern California, Los Angeles, CA; Division of Statistical Genomics, Department of Genetics, Washington University School of Medicine, St. Louis, MO; Clinical and Translational Epidemiology Unit, Mongan Institute, Massachusetts General Hospital, Boston, MA; Graduate School of Data Science, Seoul National University, Seoul; University of Mississippi Medical Center, Jackson, MS; Human Genetics Center, Department of Epidemiology, The University of Texas Health Science Center at Houston School of Public Health, Houston, TX; Survey Research Center, Institute for Social Research, University of Michigan, Ann Arbor, MI; Department of Computational Biology, F-75015 Paris, France Institut Pasteur, Université Paris Cité, Paris; Cardiovascular Health Research Unit, Department of Medicine, University of Washington, Seattle, WA; Center for Biostatistics and Data Science, Institute for Informatics, Data Science, and Biostatistics, Washington University in St. Louis, School of Medicine, St. Louis, MO; Department of Biostatistics, Vanderbilt University Medical Center, Nashville, TN; Division of Statistical Genomics, Department of Genetics, Washington University School of Medicine, St. Louis, MO; Icelandic Heart Association, Kopavogur; The Institute for Translational Genomics and Population Sciences, Department of Pediatrics, The Lundquist Institute for Biomedical Innovation at Harbor-UCLA Medical Center, Torrance, CA; Lothian Birth Cohorts, Department of Psychology, The University of Edinburgh, Edinburgh; Laboratory for Pharmacogenomics, RIKEN Center for Integrative Medical Sciences, Yokohama, Kanagawa; Department of Epidemiology, Tulane University School of Public Health and Tropical Medicine, New Orleans, LA; Department of Data Sciences, Hunter Medical Research Institute, New Lambton Heights, NSW; Finnish Cardiovascular Research Center – Tampere, Department of Clinical Chemistry, Fimlab Laboratories and Faculty of Medicine and Health Technology, Tampere University, Tampere; Department of Psychiatry, Amsterdam UMC/Vrije universiteit, Amsterdam; Department of Medicine, Surgery and Health Sciences, University of Trieste, Trieste; Department of Medicine, Surgery and Health Sciences, University of Trieste, Trieste; Department of Molecular Biology and Biochemistry, Medical University Graz, Graz, Styria; Laboratory of Epidemiology and Population Sciences, Health Disparities Research Section, National Institute on Aging, National Institutes of Health, Baltimore, MD; Division of Sleep and Circadian Disorders, Department of Medicine, Brigham and Women’s Hospital, Boston, MA; Department of Biostatistics, University of North Carolina at Chapel Hill, Chapel Hill, NC; Saw Swee Hock School of Public Health, National University of Singapore and National University Health System, Singapore; Department of Biostatistics, Boston University School of Public Health, Boston, MA; Institut universitaire de cardiologie et de pneumologie de Québec-Université Laval, Department of Molecular Biology, Medical Biochemistry and Pathology, Université Laval, Quebec City, QC; Department of Epidemiology, University of Groningen, University Medical Center Groningen, Groningen; Survey Research Center, Institute for Social Research, University of Michigan, Ann Arbor, MI; Interfaculty Institute for Genetics and Functional Genomics, Department of Functional Genomics, University Medicine Greifswald, Greifswald; Beijing Institute of Ophthalmology, Beijing Tongren Hospital, Capital Medical University, Beijing Ophthalmology and Visual Sciences Key Laboratory, Beijing, Beijing; School of Public Health and Preventive Medicine, Monash University, Melbourne, VIC; Survey Research Center, Institute for Social Research, University of Michigan, Ann Arbor, MI; Department of Data Science, University of Mississippi Medical Center, Jackson, MS; Jackson Heart Study, University of Mississippi Medical Center, Jackson, MS; School of Medicine and Public Health, College of Health Medicine and Wellbeing, University of Newcastle, New Lambton Heights, NSW; Department of Epidemiology, Tulane University School of Public Health and Tropical Medicine, New Orleans, LA; Cardiovascular Health Research Unit, Department of Medicine, University of Washington, Seattle, WA; Division of Genetic Medicine, Department of Medicine, Vanderbilt University Medical Center, Nashville, TN; Division of Sleep and Circadian Disorders, Department of Medicine, Brigham and Women’s Hospital, Boston, MA; Center for Research on Genomics and Global Health, National Human Genome Research Institute, National Institutes of Health, Bethesda, MD; Survey Research Center, Institute for Social Research, University of Michigan, Ann Arbor, MI; Lothian Birth Cohorts, Department of Psychology, The University of Edinburgh, Edinburgh; Lothian Birth Cohorts, Department of Psychology, The University of Edinburgh, Edinburgh; German Center for Cardiovascular Research (DZHK), partner site Greifswald, Greifswald; Department of Biostatistics, University of North Carolina at Chapel Hill, Chapel Hill, NC; Division of Epidemiology, Department of Medicine, Vanderbilt University Medical Center, Nashville, TN; Division of Sleep and Circadian Disorders, Department of Medicine, Brigham and Women’s Hospital, Boston, MA; Survey Research Center, Institute for Social Research, University of Michigan, Ann Arbor, MI; Cardiovascular Health Research Unit, Department of Medicine, University of Washington, Seattle, WA; Department of Epidemiology, School of Public Health, University of Washington, Seattle, WA; Department of Biomedical Informatics, Vanderbilt University Medical Center, Nashville, TN; Cardiovascular Medicine, Department of Medicine, University of Wisconsin School of Medicine and Public Health, Madison, Wisconsin; Cardiovascular Disease (CVD) Genetic Epidemiology Laboratory, Department of Epidemiology, University of North Carolina at Chapel Hill, Chapel Hill, NC; Department of Neurology, Medical University Graz, Graz, Styria; Interfaculty Institute for Genetics and Functional Genomics, Department of Functional Genomics, University Medicine Greifswald, Greifswald; Department of Epidemiology, School of Public Health, University of Michigan, Ann Arbor, MI; Beijing Institute of Ophthalmology, Beijing Tongren Hospital, Capital Medical University, Beijing Ophthalmology and Visual Sciences Key Laboratory, Beijing, Beijing; Finnish Cardiovascular Research Center – Tampere, Department of Clinical Physiology, Tampere University Hospital and Faculty of Medicine and Health Technology, Tampere University, Tampere; Department of Epidemiology, School of Public Health, University of Michigan, Ann Arbor, MI; Department of Epidemiology, School of Public Health, University of Michigan, Ann Arbor, MI; Laboratory of Epidemiology and Population Sciences, Intramural Research Program, National Institute on Aging, National Institutes of Health, Baltimore, MD; Population Sciences Branch, Division of Intramural Research, National Heart, Lung, and Blood Institute, National Institutes of Health, Bethesda, MD; Metabolism Program, Broad Institute of MIT and Harvard, Cambridge, MA; Department of Cardiology, George Washington University, Washington, DC; Institute of Medical Science, The University of Tokyo, Minato-ku, Tokyo; School of Public Health and Preventive Medicine, Monash University, Melbourne, VIC; Department of Epidemiology, University of Groningen, University Medical Center Groningen, Groningen; Department of Psychiatry, Fujita Health University School of Medicine, Toyoake, Aichi; Department of Genetics, University of North Carolina, Chapel Hill, NC; Centre for Population Health Research, Department of Clinical Physiology and Nuclear Medicine, InFLAMES Research Flagship, Turku University Hospital and University of Turku, Turku; Faculty of Medical Sciences , Institute for Laboratory Medicine, Private University in the Principality of Liechtenstein, Vaduz; Central Laboratory, Cantonal Hospital Graubünden, Chur; Urban Health Collaborative, Department of Epidemiology and Biostatistics, Drexel University, Philadelphia, PA; Department of Nutritional Sciences, University of Michigan, Ann Arbor, MI; Lothian Birth Cohorts, Department of Psychology, The University of Edinburgh, Edinburgh; Department of Psychiatry, Fujita Health University School of Medicine, Toyoake, Aichi; Division of Epidemiology and Community Health, School of Public Health, University of Minnesota, Minneapolis, MN; School of Medicine and Public Health, College of Health Medicine and Wellbeing, University of Newcastle, New Lambton Heights, NSW; Division of General Internal Medicine and Population Science, Heersink School of Medicine, University of Alabama at Birmingham, Birmingham, AL; Survey Research Center, Institute for Social Research, University of Michigan, Ann Arbor, MI; Department of Epidemiology, University of Groningen, University Medical Center Groningen, Groningen; Department of Medicine, Surgery and Health Sciences, University of Trieste, Trieste; Saw Swee Hock School of Public Health, National University of Singapore and National University Health System, Singapore; Lothian Birth Cohorts, Department of Psychology, The University of Edinburgh, Edinburgh; The Institute for Translational Genomics and Population Sciences, Department of Pediatrics, The Lundquist Institute for Biomedical Innovation at Harbor-UCLA Medical Center, Torrance, CA; Department of Medicine, Surgery and Health Sciences, University of Trieste, Trieste; Saw Swee Hock School of Public Health, National University of Singapore and National University Health System, Singapore; Department of Epidemiology, University of Groningen, University Medical Center Groningen, Groningen; Beijing Tongren Eye Center, Beijing Tongren Hospital, Capital Medical University, Beijing, Beijing; Department of Epidemiology, University of Groningen, University Medical Center Groningen, Groningen; The Institute for Translational Genomics and Population Sciences, Department of Pediatrics, The Lundquist Institute for Biomedical Innovation at Harbor-UCLA Medical Center, Torrance, CA; Cardiovascular Disease (CVD) Genetic Epidemiology Laboratory, Department of Epidemiology, University of North Carolina at Chapel Hill, Chapel Hill, NC; Department of Epidemiology, Tulane University School of Public Health and Tropical Medicine, New Orleans, LA; Laboratory of Epidemiology and Population Sciences, Health Disparities Research Section, National Institute on Aging, National Institutes of Health, Baltimore, MD; Institute of Medical Science, The University of Tokyo, Minato-ku, Tokyo; Roden; Institute for Maternal and Child Health – IRCCS “Burlo Garofolo”, Trieste; Population Health Research Institute, Department of Medicine, McMaster University, Hamilton, ON; Lothian Birth Cohorts, Department of Psychology, The University of Edinburgh, Edinburgh; Laboratory of Epidemiology and Population Sciences, Health Disparities Research Section, National Institute on Aging, National Institutes of Health, Baltimore, MD; Jackson Heart Study, University of Mississippi Medical Center, Jackson, MS; Center for Biostatistics and Data Science, Institute for Informatics, Data Science, and Biostatistics, Washington University in St. Louis, School of Medicine, St. Louis, MO; Division of Epidemiology, Department of Medicine, Vanderbilt University Medical Center, Nashville, TN; Department of Medicine, Surgery and Health Sciences, University of Trieste, Trieste; Department of Psychiatry and Psychotherapy, University Medicine Greifswald, Greifswald, Mecklenburg-Western Pomerania; Center for Biostatistics and Data Science, Institute for Informatics, Data Science, and Biostatistics, Washington University in St. Louis, School of Medicine, St. Louis, MO; Icelandic Heart Association, Kopavogur; Department of Epidemiology, School of Public Health, University of Michigan, Ann Arbor, MI; School of Medicine and Public Health, College of Health Medicine and Wellbeing, University of Newcastle, New Lambton Heights, NSW; Rothschild Foundation Hospital, Institut Français de Myopie, Paris; School of Public Health and Preventive Medicine, Monash University, Melbourne, VIC; Graduate School of Data Science, Seoul National University, Seoul; Finnish Cardiovascular Research Center – Tampere, Department of Clinical Chemistry, Fimlab Laboratories and Faculty of Medicine and Health Technology, Tampere University, Tampere; Department of Epidemiology, Tulane University School of Public Health and Tropical Medicine, New Orleans, LA; Department of Biostatistics, Boston University School of Public Health, Boston, MA; Human Genetics Center, Department of Epidemiology, The University of Texas Health Science Center at Houston School of Public Health, Houston, TX; Cardiovascular Disease (CVD) Genetic Epidemiology Laboratory, Department of Epidemiology, University of North Carolina at Chapel Hill, Chapel Hill, NC; Department of Psychiatry, Amsterdam UMC/Vrije universiteit, Amsterdam; Department of Epidemiology, School of Public Health, University of Michigan, Ann Arbor, MI; Division of Statistical Genomics, Department of Genetics, Washington University School of Medicine, St. Louis, MO; Cardiovascular Health Research Unit, Department of Medicine, University of Washington, Seattle, WA; Division of Sleep and Circadian Disorders, Department of Medicine, Brigham and Women’s Hospital, Boston, MA; Department of Clinical Epidemiology, Leiden University Medical Center, Leiden; Center for Research on Genomics and Global Health, National Human Genome Research Institute, National Institutes of Health, Bethesda, MD; The Institute for Translational Genomics and Population Sciences, Department of Pediatrics, The Lundquist Institute for Biomedical Innovation at Harbor-UCLA Medical Center, Torrance, CA; Department of Neurology, Medical University Graz, Graz, Styria; Saw Swee Hock School of Public Health, National University of Singapore and National University Health System, Singapore; The Clinical Research Center at Shizuoka General Hospital, Shizuoka; Survey Research Center, Institute for Social Research, University of Michigan, Ann Arbor, MI; Department of Population and Quantitative Health Sciences, Case Western Reserve University, Cleveland, Ohio; Department of Epidemiology, University of North Carolina at Chapel Hill, Chapel Hill, NC; Division of Endocrinology, Diabetes and Nutrition, Department of Medicine, University of Maryland School of Medicine, Baltimore, MD; Division of Cardiovascular Science, Epidemiology Branch, National Heart, Lung, and Blood Institute, National Institutes of Health, Bethesda, MD; Division of Sleep and Circadian Disorders, Department of Medicine, Brigham and Women’s Hospital, Boston, MA; Metabolism Program, Broad Institute of MIT and Harvard, Cambridge, MA; Clinical Pharmacology and Precision Medicine, Queen Mary University of London, London; Center for Biostatistics and Data Science, Institute for Informatics, Data Science, and Biostatistics, Washington University in St. Louis, School of Medicine, St. Louis, MO; Human Genetics Center, Department of Epidemiology, The University of Texas Health Science Center at Houston School of Public Health, Houston, TX; Division of Biostatistics, Department of Population and Public Health Sciences, University of Southern California, Los Angeles, CA; Department of Psychiatry, Washington University School of Medicine, St. Louis, MO; Department of Genetic Epidemiology, University of Regensburg, Regensburg; Brown Foundation Institute of Molecular Medicine, The University of Texas Health Science Center at Houston, McGovern Medical School, Houston, TX

## Abstract

**Background:**

Gene-environment interactions may enhance our understanding of hypertension. Our previous study highlighted the importance of considering psychosocial factors in gene discovery for blood pressure (BP) but was limited in statistical power and population diversity. To address these challenges, we conducted a multi-population genome-wide association study (GWAS) of BP accounting for gene-depressive symptomatology (DEPR) interactions in a larger and more diverse sample.

**Results:**

Our study included 564,680 adults aged 18 years or older from 67 cohorts and 4 population backgrounds (African (5%), Asian (7%), European (85%), and Hispanic (3%)). We discovered seven novel gene-DEPR interaction loci for BP traits. These loci mapped to genes implicated in neurogenesis (*TGFA, CASP3*), lipid metabolism (*ACSL1*), neuronal apoptosis (*CASP3*), and synaptic activity (*CNTN6, DBI*). We also identified evidence for gene-DEPR interaction at nine known BP loci, further suggesting links between mood disturbance and BP regulation. Of the 16 identified loci, 11 loci were derived from African, Asian, or Hispanic populations. Post-GWAS analyses prioritized 36 genes, including genes involved in synaptic functions (*DOCK4, MAGI2*) and neuronal signaling (*CCK, UGDH, SLC01A2*). Integrative druggability analyses identified 11 druggable candidate gene targets, including genes implicated in pathways linked to mood disorders as well as gene products targeted by known antihypertensive drugs.

**Conclusions:**

Our findings emphasize the importance of considering gene-DEPR interactions on BP, particularly in non-European populations. Our prioritized genes and druggable targets highlight biological pathways connecting mood disorders and hypertension and suggest opportunities for BP drug repurposing and risk factor prevention, especially in individuals with DEPR.

## Background

Hypertension and high blood pressure (BP) are major risk factors for cardiovascular disease, stroke, chronic kidney disease, and vascular dementia, significantly contributing to global morbidity and mortality [1]. Despite the widespread availability of effective anti-hypertensive medications, the prevalence of hypertension has doubled worldwide over the past three decades and is projected to affect 1.6 billion individuals by 2025 [2]. Moreover, while the age-adjusted prevalence of hypertension has declined in some regions, global disparities in hypertension rates have widened [3, 4].

Genetic and environmental factors can independently increase the risk of hypertension, but gene-environment interaction (GxE) may provide a more comprehensive understanding of the genetic contributions to the disease [5–7]. A recent genome-wide association study (GWAS) of BP identified a total of 2,103 independent genetic signals, which accounts for approximately 60% of the heritability of BP [8]. Consequently, a substantial portion of heritability remains unexplained. Incorporating GxE in genetic analyses of BP may yield additional information about its genetic architecture and provide new avenues to improve health by more precisely characterizing risk of high BP in the context of potentially modifiable environmental, lifestyle, and behavioral risk factors [9].

The influence of psychosocial factors on BP level is well known [10–12]. Psychosocial stress increases the incidence of hypertension, and is associated with poor hypertension control, unhealthy lifestyle behaviors, and non-compliance with treatment regimens [13]. The relationship between depressive symptoms and BP is complex. While some studies have shown an association of depressive symptoms with incidence of hypertension [14], others have reported an association of depressive symptoms with lower BP levels [15]. Whilst, a recent study provided evidence of depression as a causal risk factor of hypertension using Mendelian Randomization [16]. Our previous study examined the effect modification of genetic factors by dichotomous psychosocial factors on BP in up to 128,894 individuals [17]. This highlighted the significance of gene-psychosocial factors interactions in gene discovery for BP, especially among individuals of African ancestry. However, the statistical power and population diversity of the study were limited. To address these shortcomings, we increased the sample size up to five-fold by incorporating now available biobank data. In addition, we defined psychosocial exposures as both dichotomous and quantitative, potentially improving the statistical power to identify novel findings. We report genome-wide association meta-analyses of systolic BP (SBP), diastolic BP (DBP), and pulse pressure (PP) in the context of depressive symptomatology (DEPR) in a sample of up to 564,680 participants from populations of African (AFR), Asian (ASN), European (EUR), and Hispanic (HIS) backgrounds.

## Results

### Overview

**Figure 1:**
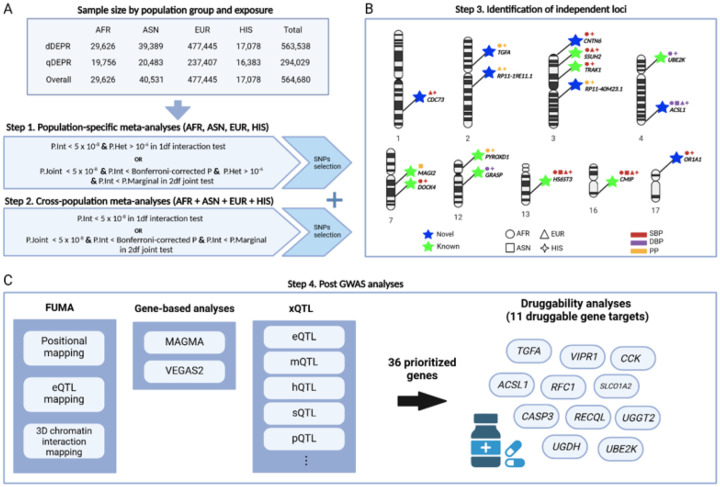
Study overview Created in BioRender. Lee, S. (2024) BioRender.com/a15i893 **A**. For each BP trait, association analyses were conducted accounting for SNP × depressive symptomatology (DEPR) interaction effects using two exposures: dichotomous DEPR (dDEPR) and quantitative (qDEPR). For each population group, study-specific results were combined to perform 1df interaction test and 2df joint test. Population-specific meta-analyses were carried out separately for each group: African (AFR), Asian (ASN), European (EUR), and Hispanic (HIS) and subsequently combined for cross-population meta-analyses. **B**. A total of 16 independent loci were identified through SNP × DEPR interaction effects, including seven novel and nine known loci for BP. **C**. Gene prioritization was performed using FUMA, gene-based analyses, and xQTL. Druggability analyses of 36 prioritized genes identified 11 druggable gene targets.

A total of 564,680 individuals from four populations were included in the study, comprising 85% EUR, 7% ASN, 5% AFR, and 3% HIS. Descriptive statistics are provided in **Supplemental Table 1**. Because the quantitative DEPR exposure was not available in some biobanks, sample sizes were larger for dichotomous DEPR (dDEPR) than quantitative DEPR (qDEPR). As shown in Fig. 1, the dDEPR analyses included 563,538 individuals after excluding two studies where the number of individuals with DEPR (N_exp_) was less than 10 (**Supplemental Table 2**). Among individuals with dDEPR, 15% had DEPR on average. The qDEPR analyses consisted of 294,029 participants from EUR (80%), ASN (7%), AFR (7%), and HIS (6%) populations.

### dDEPR analyses

We identified nine independent loci that showed evidence of association with BP traits modified by dDEPR in cross-population meta-analyses (CPMA) or population-specific meta-analyses (Table 1). Of these, three loci tagged by rs1664073690 (1q31.3), rs10178576 (2q13.3), and rs113521945 (4q35.1) were novel. The other six loci tagged by rs115760284 (3p22.1), rs147967138 (7q21.11), rs757194 (7q31.1), rs7979305 (12p12.1), rs75095906 (13q32.1), and rs9931605 (16q23.2) were previously reported for BP (**Supplemental Table 3**). Eight of the nine loci were identified via the 1df interaction test (P.Int < 5 × 10^− 8^) (Table 1). In the 2df joint test, a total of 904 loci were associated with at least one BP trait (350 loci were associated with SBP, 337 loci were associated with DBP, and 364 loci were associated with PP). Among them, one previously reported BP locus (rs757194 on 7q31.1) showed evidence of association with SBP through interaction with dDEPR using the specified criteria (P.Joint = 7.99 × 10^− 9^; P.Int = 1.39 × 10^− 7^).

**Figure 2:**
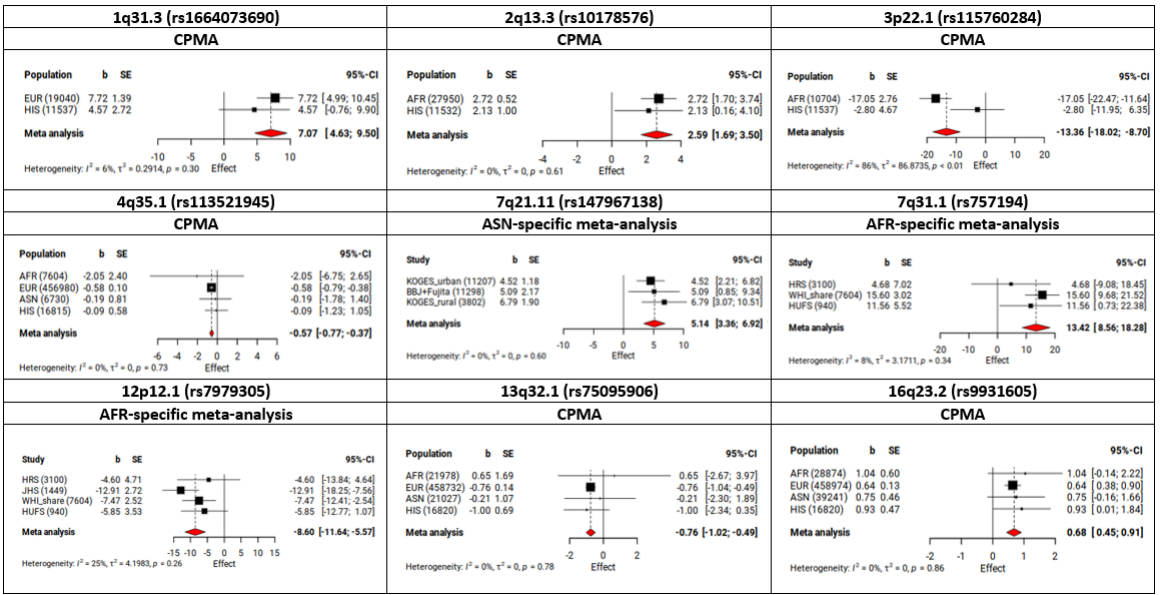
Forest plots of interaction effects at novel and known loci identified in the dDEPR analyses CPMA, cross-population meta-analyses; AFR, African; ASN, Asian, EUR, European; HIS, Hispanic; b, the interaction effects estimated in the 1df interaction test (Effect is in mmHg); SE, standard error of interaction effects estimated in the 1df interaction test; CI, confidence interval Black squares and error bars represent the effect size and its 95% CI for each population in CPMA or for each study in population-specific meta-analyses. Red diamond represents the overall effect size calculated in the meta-analysis where the center indicates the point estimate and its edges represent 95% CI of the estimate.

The three top single nucleotide polymorphisms (SNPs) at novel loci (1q31.3, 2q13.3, and 4q35.1) were identified in the CPMA and showed no evidence of heterogeneity across population groups (P.Het > 0.003) (Table 1). Two of them were common variants with minor allele frequency (MAF) greater than 0.05 in at least one population group while one (rs1664073690 on 1q31.3) had a low frequency (MAF = 0.02). This variant was present at low frequency in EUR and HIS but was absent in both ASN and AFR. rs10178576 (2q13.3) was common in AFR (MAF = 0.11) but was not observed in either ASN or EUR populations (Fig. 2). rs113521945 (4q35.1) was observed across all four population groups. While a significant interaction was observed only in EUR, the direction of the effect was consistent across all four groups (Fig. 2).

Among the six top SNPs at known BP loci (3p22.1, 7q21.11, 7q31.1, 12p12.1, 13q32.1, and 16q23.2), four SNPs on 3p22.1, 7q21.11, 7q31.1, and 12p12.1 showed the most significant associations or were exclusively observed in non-EUR populations (Fig. 2). Notably, three of them (rs115760284 on 3p22.1, rs757194 on 7q31.1, and rs7979305 on 12p12.1) were absent in both EUR and ASN but were present at low frequency in AFR (0.01 ≤ MAF ≤ 0.05) and were rare in HIS (MAF < 0.01) (Table 1). Interestingly, rs115760284 (3p22.1) showed some heterogeneity between AFR and HIS (I^2^ > 80%, P.Het < 0.01), with a greater effect size in AFR (Fig. 2). Moreover, a locus on 7q21.11 was detected solely in ASN population among 26,307 individuals, with no evidence of heterogeneity across ASN studies (P.Het > 0.003). Two loci tagged by rs75095906 (13q32.1) and rs9931605 (16q23.2) were identified in the CPMA analyses, with no evidence of heterogeneity by population group. Across all nine top SNPs identified in the dDEPR analyses, no evidence of sex heterogeneity was observed. However, some SNPs could not be evaluated due to a limited sample size in males passing QC.

### qDEPR analyses

We identified seven independent loci that showed evidence of association with BP traits modified by qDEPR in CPMA or population-specific meta-analyses (Table 2). Four loci tagged by rs77572777 (2q14.2), rs148780833 (3p26.3), rs748650739 (3q13.11), and rs140618249 (17p13.3) were novel. The other three loci tagged by rs59284269 (3p25.3), rs145132348 (4p14), and rs114544309 (12q13.13) were previously reported for BP (**Supplemental Table 3**). Five loci, including two novel, were identified using the 1df interaction test (P.Int < 5 × 10^− 8^) (Table 2). In the 2df joint test, a total of 316 loci were associated with at least one BP trait (144 loci were associated with SBP, 160 loci were associated with DBP, and 157 loci were associated with PP). Among them, two novel loci tagged by rs77572777 (2q14.2) and rs748650739 (3q13.11) were associated with PP through interaction with qDEPR. Notably, two of the novel loci rs148780833 (3p26.3) and rs140618249 (17p13.3) identified in the 1df test (P.Int < 5 × 10^− 8^) also showed evidence of an association with SBP through interaction with qDEPR using the 2df joint test (P.Joint < 5 × 10^− 8^).

The four top SNPs tagging the novel loci include rs77572777 (2q14.2) and rs748650739 (3q13.11) from the HIS-specific analyses, and rs148780833 (3p26.3) and rs140618249 (17p13.3) from the CPMA. None of these four SNPs showed evidence of heterogeneity across populations or studies (P.Het > 0.003) and all were of low frequency (MAF = 0.01–0.02). Except for rs77572777 on 2q14.2, the three other SNPs were polymorphic only in AFR and HIS populations.

**Figure 3:**
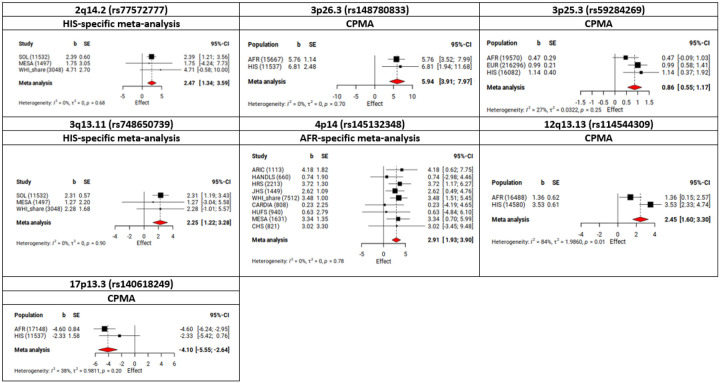
Forest plots of interaction effects at novel and known loci identified in the qDEPR analyses CPMA, cross-population meta-analyses; AFR, African; ASN, Asian, EUR, European; HIS, Hispanic; b, the interaction effects estimated in the 1df interaction test (Effect is in mmHg); SE, standard error of interaction effects estimated in the 1df interaction test; CI, confidence interval Black squares and error bars represent the effect size and its 95% CI for each population in CPMA or for each study in population-specific meta-analyses. Red diamond represents the overall effect size calculated in the meta-analysis where the center indicates the point estimate and its edges represent 95% CI of the estimate.

Among the three known loci (3p25.3, 4p14, and 12q13.13) identified in the qDEPR analyses, two loci on 4p14 and 12q13.13 were not observed in EUR population. Of these two, the 4p14 locus tagged by rs145132348 and identified in AFR-specific analyses showed no heterogeneity across AFR studies contributing to the meta-analyses in this population (Fig. 3). The other locus on 12q13.13 tagged by rs114544309 and identified in CPMA showed the most significant association in HIS, with some evidence of heterogeneity between AFR and HIS and a greater effect size in HIS (Fig. 3). rs59284269 (3p25.3) identified in CPMA showed no evidence of heterogeneity by population group. No evidence of sex heterogeneity was observed across all seven top SNPs identified in the qDEPR analyses.

#### Comparison between dDEPR and qDEPR analyses

Of the 16 identified loci (Tables 1 and 2), four top SNPs (rs77572777, rs148780833, rs748650739, and rs114544309) were identified exclusively in the qDEPR analyses (**Supplemental Figs. 1 and 2**). This appears to largely reflect differences in included studies in the two types of analyses (**Supplemental Fig. 3**). In the CPMA, approximately 15 million SNPs were included in the dDEPR and 21 million SNPs in the qDEPR analyses. Notably, nearly 6 million SNPs were analyzed only in the qDEPR analyses, mainly because they were filtered out in the dDEPR analyses by the stringent study-level filters. Conversely, fewer than half a million SNPs were analyzed exclusively in dDEPR analyses, likely due to some large biobank samples where only dichotomous exposure was available while the quantitative exposure was not. The four SNPs identified only in qDEPR analyses were filtered out of the dDEPR analyses during study-level QC (rs148780833) or at the meta-analysis QC because they were present in only one study (rs77572777 and rs748650739), or in only one population (rs114544309) (**Supplemental Figs. 2**). The remaining 12 loci were present in both dDEPR and qDEPR analyses. As illustrated in **Supplemental Figs. 1 and 2**, there was a remarkable consistency between the two analyses even though magnitude of effects and statistical significance varied between them.

#### Gene-based and pathway analyses

Using 1df interaction test results, Multi-marker Analysis of GenoMic Annotation (MAGMA) and Versatile Gene-Based Association Study 2 (VEGAS2) ranked genes and pathways based on the combined association of SNPs within a gene with BPs. Both MAGMA and VEGAS2 gene-based tests identified a gene-wide significant association for GLTPD2, with similar results observed for several other genes among the top 20 genes (**Supplemental Table 4**). An additional gene, TMEM199, was discovered by VEGAS2. These two genes were not identified at genome-wide significance in the GWAS. Pathway analyses suggest DEPR-specific genetic pathways that influence BP, including retinoid signaling, remodeling of acyl chains of phosphatidylethanolamine, nucleotide-binding oligomerization domain containing 2 (NOD2) protein signaling, and response to stress (**Supplemental Table 5**).

#### Functional annotation and gene prioritization

Functional annotation was conducted for all SNPs in linkage disequilibrium (LD) (r^2^ > 0.4) with the top SNPs tagging all identified novel and known BP loci. All the top SNPs were annotated as either intergenic or intronic variants, suggesting a potential role for regulatory mechanisms. Among 31 genes identified by FUMA, six genes were predicted to be highly intolerant to loss-of-function mutation based on probability of loss-of-function intolerance (pLI) score > 0.9, including *CMIP, ZBTB47, DOCK4, UBE2K, PDS5A*, and *GRASP* (**Supplemental Table 6**). Multiple genes exhibited high CADD scores (> 12.37) among SNPs in LD, suggesting potential deleterious effects. Three additional genes were identified through associations with various quantitative trait loci (xQTL), which include *CASP3, DBI*, and *UGGT2*. Details on functional annotations are described in **Supplemental Table 7**.

A total of 36 genes were prioritized by functional annotations of both novel and known loci, as well as gene-based analyses. These prioritized genes showed enrichment of gene expression in the brain and whole blood (**Supplemental Fig. 4**). Additionally, they demonstrated evidence of enrichment in two pathways involved in myogenesis and immune system in dendrite cells, as well as enrichment in four potential microRNA regulatory targets (**Supplemental Table 8**).

#### Druggability analyses

We investigated the potential druggability of the identified 36 candidate gene product targets using an integrative approach as previously described [18]. We queried dDEPR and qDEPR exposure candidate gene targets using the Drug-Gene Interaction database (DGIdb), which identified 11 genes annotated as members of the druggable genome (**Supplemental Table 9**). Several of these gene targets are implicated in metabolic pathways (*ACSL1, DBI, UGDH, SLCO1A2*), vascular wall signaling (*TGFA, CAV3, SSUH2, DOCK4*), DNA damage response or apoptosis (*CASP3, RFC1, RECQL*), and neuroactive ligand-receptor interaction (*VIPR1, CCK*). We identified 11 genes with FDA approved drug interactions that have been evaluated in late-stage clinical trials using DrugBank, ChEMBL, and ClinicalTrials.gov databases (**Supplemental Table 10**). Two of these gene targets (*CASP3* and *UGDH*) were identified as targets of aspirin, a well-established and safe drug used to treat pain, inflammation, and reduce cardiovascular events. *UBE2K* was identified as a target of the central nervous system stimulant, dextroamphetamine, used to treat attention-deficit disorder (ADHD) and narcolepsy, however its use has been federally controlled due to the high potential for abuse. *CCK* was also identified as a target of the vasodilator, diazoxide, which is used to manage hypoglycemia due to pancreatic cancer or other conditions. Several genes (*CCK, SLCO1A2, UGGT2*) were identified as targets of drugs (diazoxide, nadolol, hydrochlorothiazide) used to treat hypertension, suggesting opportunities for drug repositioning and risk factor prevention.

## Discussion

In this large-scale genome-wide interaction study, we identified 16 genetic loci whose association with BP was modified by DEPR defined as a dichotomous or a quantitative exposure. These data provide support for molecular mechanisms connecting DEPR and BP and highlight several genes as possible druggable targets with clinical potential for BP regulation in individuals with DEPR.

Nearly 70% of our findings were derived from non-EUR populations, likely due to differences in allele frequency across populations and/or to population differences in SNP × DEPR interaction effect sizes. Notably, several of the identified SNPs were monomorphic in EUR. Variations in MAF across population groups have been shown to contribute to differences in disease prevalence across populations [19]. The risk of hypertension varies considerably across populations, being more prevalent in AFR and HIS populations [20, 21]. More than half of our findings come from AFR and/or HIS. AFR populations generally exhibit greater genetic diversity and more pronounced allele frequency differences compared to other populations [22]. Self-identified HIS populations in the US include admixed individuals with varying proportions of EUR, AFR, and Amerindian genetic backgrounds, adding further complexity. Interestingly, patterns of associations were similar in AFR and HIS populations at several loci near the genes *TGFA, TRAK1, CNTN6*, and *OR1A1*. GWAS of BP have identified differences in BP loci by population groups, while partial generalization of BP loci between populations has also been reported [23–25]. Thus, there is a critical need for expanding genetic studies of BP in non-EUR populations. In our study, among nine known BP loci identified with evidence for gene-DPER interaction, six loci (3p22.1, 7q21.11, 7q31.1, 12p12.1, 4p14, and 12q13.13) were derived from non-EUR populations while they were previously discovered as BP loci in EUR population. This further underscores the importance of considering DEPR effect modification on BP for diverse populations. Multiple studies have shown mixed results regarding the association between depressive symptomatology and hypertension [14, 26, 27]. Despite this variability, depression has been consistently linked to an increased risk of cardiovascular morbidity and mortality [28]. Typically, depression arises in response to stressful events, and stress is a major risk factor for hypertension [29]. Both hypertension and depression show higher prevalence among individuals of non-EUR populations, highlighting significant racial and ethnic disparities [20, 21, 30, 31].

Functional annotation of the novel loci revealed genes implicated in neurogenesis, lipid metabolism, neuronal apoptosis, and synaptic activity. A locus on chromosome 2 mapped to an intron of the *TGFA* gene, which encodes a ligand for the epidermal growth factor receptor and plays a crucial role in neural cell proliferation and differentiation [32, 33]. Previous studies suggested *TGFA*’s role in neurogenesis and angiogenesis in adult injured brain and the immune system [34, 35]. Furthermore, genetic variants in *TGFA* have been associated with response to antidepressant treatment in GWAS [36, 37]. *ACSL1* encodes an isozyme of the long-chain fatty-acid-coenzyme A ligase family, which operates in lipid biosynthesis and fatty acid degradation. Animal models have demonstrated that *ACSL1* modulates lipid metabolism, inflammation, and oxidative stress in kidney disease [38, 39]. In fact, the kidney plays a critical role in BP regulation [40]. The *ACSL1* locus was associated with DNA methylation levels (mQTL) of ACSL1 in blood. Functional annotations of this novel locus also highlight several additional genes, including *CASP3. CASP3* encodes a cysteine-aspartic acid protease (Caspase-3) that plays a critical role in neuronal apoptosis, neurogenesis, and synaptic activity [41–44]. Notably, the *ACSL1* locus was associated with the splicing event of *CASP3* in brain tissue. Interestingly, a recent study highlighted the role of Caspase-3 in pathogenesis of depressive disorders [45]. *CNTN6* encodes Contactin-6, a neuronal cell adhesion molecule that facilitates neurite outgrowth and synaptogenesis [46]. Mutations in this gene increase the risk for autism spectrum disorders [47]. *DBI* encodes a diazepam binding inhibitor, which is regulated by hormones and acts as a neuropeptide in brain synapses [48]. Our results showed an intergenic variant (rs77572777 on 2q14.2) with an expression quantitative trait locus (eQTL) of *DBI* in brain tissue. A previous study reported that *DBI* expression in the brain decreased with long-term social isolation stress [49]. An increased level of the protein encoded by *DBI* has been suggested as a prognostic value in cardiovascular disease [50].

Several known loci for BP were identified through interactions with DEPR in our study and implicated several genes previously reported to be associated with mental disorders. These genes include *DOCK4, HS6ST3*, and *MAGI2*. The *DOCK4* locus was associated with SBP in the AFR population. DOCK4 is a member of the dedicator of cytokinesis family and is involved in cell migration [51]. Animal models have suggested a role of *DOCK4* in excitatory synaptic transmission and social behavior [52]. Variants in *DOCK4* have been associated with response to antidepressants, autism spectrum disorder, and schizophrenia [53, 54]. A recent GWAS of stress-induced vasomotion identified an association with variants in *DOCK4*, which were also linked to an increased risk of adverse cardiovascular events [55]. *HS6ST3* encodes heparin sulfate sulfotransferases involved in proliferation, inflammation, and blood coagulation. Variants within or near this gene have been associated with schizophrenia, major depressive disorder, and coronary artery calcified atherosclerotic plaque [56–58]. *MAGI2* encodes a synaptic scaffolding molecule and shows high expression in the brain and postsynaptic density area of spine [59]. In our data, the MAGI2 locus was observed only in ASN population, and variants in this gene have been associated with depressive symptoms in an East Asian cohort as well as in other population groups [60–62].

Our druggability analyses suggest potential opportunities for drug repurposing and risk factor prevention. The identified genes include CASP3 and UGDH as targets for aspirin and CCK, SLC01A2, and UGGT2 for antihypertensive medications. UGDH encodes an integral Golgi membrane protein involved in signal transduction and cell migration. A previous study has shown its nominal association with brain electrical activity linked to psychiatric conditions including depression, and suggested that this association may be population-specific [63]. This is consistent with our finding that the associated SNP (rs145132348 on 4p14) was identified only in individuals of AFR and HIS populations. CCK encodes cholecystokinin (CCK), a digestive enzyme and a neuropeptide that regulates emotional states [64, 65]. Patients with major depression showed increased CCK levels in cerebrospinal fluid [66]. CCK enzyme also plays role in BP regulation and predicts cardiovascular mortality in elder females [67, 68]. SLCO1A2 (or OATP1A2) encodes a sodium-independent transporter that is crucial for transporting hormones across the blood-brain barrier into the central nervous system and has been suggested as a potential modulator of mood disorders [69–71]. UGGT2 encodes a soluble protein of the endoplasmic reticulum and has been associated with impulsive behaviors [72, 73]. It is important to note that some of these drug-gene interactions may also reflect the medication use for individuals with chronic depression and warrant follow-up to determine their direct impact on hypertension and cardiovascular risk [26].

Findings from our prior study [17] were generally not replicated in this study, likely due to the use of a new modeling strategy that includes additional adjustment for potential confounders. One notable exception is the reported gene-DEPR interaction at the FSTL5 locus by 2df joint test, tagged by two SNPs (rs138187213 and rs5863461). In our dDEPR analyses, both SNPs showed associations in the 1df interaction test (rs138187213, P.Int = 8.48 × 10^− 4^; rs5863461, P.Int = 2.89 × 10^− 4^). Similar results were observed in the qDEPR analyses, with both SNPs showing evidence of interactions (rs138187213, P.Int = 8.19 × 10^− 5^; rs5863461, P.Int = 1.06 × 10^− 4^).

Our study benefits from a large sample size with diverse population backgrounds, which allows for a comprehensive analysis of the interactions across different populations. Moreover, our methodological approach using two complementary definitions of DEPR sought to enhance novel discoveries. The dDEPR analyses, with a larger sample size, provided greater statistical power, while the qDEPR analyses were designed to capture subtle variations in exposure and potentially reveal associations that might have been missed in the dDEPR analyses. Notably, we observed a substantial number of SNPs analyzed in the qDEPR but not included in the dDEPR, likely due to stringent filters required for binary exposure analyses. The qDEPR analyses enabled us to identify additional loci at genome-wide level, possibly due to the assumption of linearity between the exposure and outcome being met for those specific loci. Furthermore, the consistency of associations across both analytical approaches reinforces robustness of our findings.

Several limitations should be acknowledged. First, the sample size for non-EUR population groups was relatively small compared to the EUR population, which may have limited the discovery of population-specific findings. For this reason, we combined East ASN and South ASN populations into a single population group although that may introduce heterogeneity. While combining distinct populations can introduce complexity due to underlying genetic and cultural differences, this approach was chosen to increase statistical power. Second, DEPR was captured by several different validated instruments in the participating cohorts with different sensitivities and specificities to detect depressive symptoms, which may have introduced heterogeneity and measurement error, potentially reducing statistical power. Lastly, while extensive functional annotation and druggability analyses provide biological validation/support for our findings, replication in independent samples was not possible in this study since dividing cohorts into discovery and replication analyses encountered insufficient power. Because we made extensive efforts at recruiting most of the studies known to have DEPR data, identifying suitable independent cohorts with large sample size and DEPR data availability for replication remains a major challenge. This is a particular issue for interactions identified only in non-European population groups, often in relatively modest sample sizes.

In conclusion, we identified multiple genetic loci associated with BP traits that were modified by DEPR. These data emphasize the importance of considering DEPR as an effect modifier in BP gene discovery, particularly in non-EUR populations. They also provide insights into the molecular basis of the relationships between DEPR and BP, and highlight the potential of applying such information to enhance more personalized approaches to hypertension management in individuals with DEPR.

## Methods

### Study design and participants

All participating cohorts were part of Gene-Lifestyle Interactions Working Group of the Cohorts for Heart and Aging Research in Genomic Epidemiology (CHARGE) Consortium [74]. Except for the UK Biobank, the study included adult men and women aged 18 years or older from four population groups defined based on self-reported participant’s race and ethnicity: AFR (including self-reported Black), ASN (including East Asian and South Asian), EUR (including self-reported White), and HIS. The UK Biobank used the Pan-UKB data to define population groups based on shared genetic similarity and demographic history [75]. GWAS considering the interaction between gene and DEPR were conducted within each individual study by population group. Population-specific meta-analyses were then performed using summary statistics, followed by cross-population meta-analyses based on the population-specific results (Fig. 1). All participating studies obtained written informed consent from their participants and approval from the appropriate institutional review boards. Details about the participating studies are provided in the **Supplemental Material**.

### Blood pressure (BP) traits

Three BP traits were considered as outcome variables: SBP, DBP, and PP. Pulse pressure was calculated as the difference between SBP and DBP. When multiple BP readings were taken during the same examination, the average of all SBP or DBP readings were used. For participants taking any anti-hypertensive medications, SBP and DBP values were adjusted by adding 15 mm Hg and 10 mm Hg, respectively, to the measured values [76, 77]. Extreme values for each BP variable were winsorized if they were more than six standard deviations (SDs) above or below the mean.

### Depressive symptomatology (DEPR) exposures

Each participating study collected information on DEPR using validated screening questionnaires, as detailed in **Supplemental Table 11**. Measurements of DEPR and BP were taken during the same examination. We defined two variables as exposures: dDEPR and qDEPR.

The dDEPR exposure was defined as a binary variable by dichotomizing DEPR measures using recommended standard cut off points specific to each screening instrument. Individuals with higher depressive symptom score were categorized as the exposed group and coded as E = 1. The specific cut off points used to define the dDEPR for each study are provided in **Supplemental Table 11**. Descriptive statistics on depression score are provided in **Supplemental Table 2**.

The qDEPR exposure was defined as a standardized residual after adjusting for age and sex effects within each cohort. For studies that included multiple population groups, the variable was computed separately for each population. First, DEPR scores were winsorized if a value was more than 6 SDs above or below the mean. The scores were then regressed on age, sex, and age × sex interaction in the sex-combined samples. The resulting age- and sex-adjusted residuals were standardized using the Z-score in the combined sample. Thus, in each study, the mean and SD of qDEPR were approximately 0 and 1, respectively, as shown in **Supplemental Table 12**. For the sex-stratified analyses, the same qDEPR estimates from the sex-combined group were used.

### Genotype data

Most of the participating studies performed genotyping using Illumina or Affymetrix. Imputations were primarily carried out using Trans-Omics for Precision Medicine (TOPMed) or Haplotype Reference Consortium (HRC) reference panels. Details on genotyping and imputation are presented in **Supplemental Table 13**. Before analysis, genotype data for each cohort were restricted to SNPs mapping to autosomal chromosomes, with MAF ≥ 0.1% across all samples and an imputation quality ≥ 0.3. Indels (insertions and deletions) were also included.

### Individual study statistical analyses

Each cohort performed analyses by population subgroup using two statistical models designed for different purposes. Model 1 was a joint effect model that accounts for the SNP main effect, DEPR effect, and the interaction effect between SNP and DEPR:

$$E(BP)=\beta_{0}+\beta_{SNP}SNP+\beta_{\text{DEPR}}\text{DEPR}+\beta_{SNP\times \text{DEPR}}SNP\times \text{DEPR}+\beta_{C}C$$

Where DEPR was either dDEPR or qDEPR, and **C** was a vector of covariates, including age, age^2^, sex, field centers (if relevant), and population-specific principal components, as well as any additional cohort-specific covariates, if applicable (**Supplemental Table 13**). In model 1, additional DEPR × covariate interaction terms with age, age^2^, and sex were included in the model to minimize potential false positive findings that could result from confounding effects [78]. For the sex-stratified analyses, both sex and DEPR × sex were excluded from the model. A 1 degree of freedom (1df) interaction test was performed to evaluate SNP × DEPR interaction effect alone under the null hypothesis that β_SNP×DEPR_ = 0. A 2df joint test was used to simultaneously assess the SNP main effect and SNP × DEPR interaction effects, under the null hypothesis that β_SNP_ = β_SNPxDEPR_ = 0 [79]. When both the SNP main effect and interaction effects exist, the 2df joint test typically provides more power than the 1df interaction test [79].

Model 2 was a SNP marginal effect model:

$$E(BP)=\beta_{0}+\beta_{SNP}SNP+\beta_{C}C$$

The SNP marginal P-value (P.Marginal) was used to identify SNPs with significant evidence of interaction effects by comparing P.Marginal to the 1df interaction P-value (P.Int) in Model 1. To ensure a fair comparison, we conducted a standard GWAS (Model 2) with the same covariates used in Model 1 other than the DEPR × covariate interaction terms.

Analyses excluded subjects without genotype data or with missing data for the DEPR exposure or any covariates. Each study selected one of the specialized software tools to run analyses: GEM (https://github.com/large-scale-gxe-methods/GEM), LinGxEScanR (https://github.com/USCbiostats/LinGxEScanR), or MMAP (https://github.com/MMAP/MMAP.github.io), as described in **Supplemental Table 13**. For the studies with related subjects, MMAP was used to account for familial relatedness using linear mixed models.

### Quality control of study-specific and meta-analyses results

Quality control (QC) was performed for both study-specific and meta-analyses results using EasyQC2 software (www.genepi-regensburg.de/easyqc2). For results submitted in build hg19, genomic coordinates were lifted over to build hg38. At the study-level, QC involved different SNP filters for the two exposures. For the dDEPR, SNPs were excluded if degree of freedom (DF) was less than 20 in the unexposed, exposed, or total samples. The DF was calculated as minor allele count * imputation quality score. For the qDEPR, SNPs were removed if the DF was less than 20 in the total samples. To identify systematic errors in data preparation, allele frequency (AF) discrepancy, outliers, and missing data were assessed visually through comparison of results to reference panels derived by imputation of population-specific 1000 Genomes phase 3 version 5 (p3v5) panels to the TOPMed reference panels using the TOPMed imputation server. Any resulting concerns were addressed through consultation with the contributing studies. Genomic control (GC) inflation factors were also estimated. Next, meta-level QC was performed within each population group (AFR: 18 cohorts; ASN: 8 cohorts, EUR: 36 cohorts, HIS: 5 cohorts) to assess improper transformation of BP variables, unstable numerical computation, and excessive inflation.

### Meta-analyses

Meta-analyses were performed using an inverse-variance weighted fixed-effect model for the 1df interaction test and an inverse-covariance-matrix-weighted model for the 2df joint test [80, 81]. Analyses were first conducted separately for each population group, and then the results were combined for CPMA. The primary focus was on analyses within the sex-combined group, considering three phenotypes and two exposures. For the identified loci in the sex-combined group analyses, we performed sex-stratified analyses to assess differences in GxE by sex. GC correction was applied to the population-specific meta-analyses and subsequently once more to the CPMA [80]. Quantile-quantile (QQ) plots and GC inflation factors are shown in **Supplemental Figs. 5–14**. In the 2df joint test, there were mild to moderate inflations, mainly due to the significance at previously reported loci for BP.

### Identification of independent associated loci

The EasyStrata2 software was used to prioritize the top loci among significant results identified in 1df interaction and 2df joint tests [82]. For the CPMA, SNPs had to be present in at least two population groups with a minimum sample size of 20,000 individuals. In the EUR-specific meta-analyses, SNPs were reported if they appeared in at least three studies and in at least 3,000 individuals. These criteria were relaxed for other population groups due to smaller sample size, as shown in **Supplemental Table 14**. Only SNPs with MAF greater than 1% were reported for both population-specific and cross-population meta-analyses. SNPs located within 1 Mb of the major histocompatibility complex (MHC) region were excluded.

We considered SNPs with significant evidence of DEPR interaction effects on BP as top SNPs based on the following criteria: (1) SNPs with significant 1df interaction effect (P.Int < 5 × 10^− 8^). In population-specific analyses, SNPs were also required to show no evidence of heterogeneity (P.Het > 10^− 6^); (2) SNPs with significant 2df joint effects (P.Joint < 5 × 10^− 8^), and P.Int < Bonferroni-corrected P adjusted for the number of 2df joint variants identified in the respective CPMA or population-specific subgroup (e.g, for CPMA: dDEPR: 0.05/904 = 5.53 × 10^− 5^; qDEPR: 0.05/316 = 1.58 × 10^− 4^), and P.Int < P.Marginal. False discovery rates (FDR) were also calculated using EastyStrata2.

To identify independent loci among all significant variants, we grouped the significant variants within 500-kilobase regions and identified independent loci by LD R^2^ < 0.1, using TOPMed-imputed 1000G reference panels. If variants within regions were missing in the LD panels, the most significant variant within each region was reported. The independent loci were considered novel if the SNPs are located ± 500 kb away from the known loci previously reported in BP GWAS (**Supplemental Table 15**). For the identified independent loci, we additionally examined heterogeneity of the interaction effects by sex using the results from the sex-stratified analyses. Heterogeneity of SNP × DEPR effects between men and women was tested using two-sample Z tests [83]. The significance threshold for heterogeneity tests was defined at Bonferroni-corrected threshold based on the number of the identified independent loci.

### Gene-based analyses

We performed gene-based tests on meta-analysis summary statistics for the 1df interaction results using MAGMA implemented in FUMA [84] and VEGAS2 [85]. Both tools computed gene-based p-values by considering variants within each gene. The MAGMA method utilized a multiple linear regression model [86], while VEGAS2 analyses were conducted using the ‘top10’ parameter, which selects the top 10% variants within a gene, taking into account the number of variants and LD. This approach allowed us to include SNPs with stronger signals and exclude those that might dilute the summary statistics [85]. For both MAGMA and VEGAS2, we used 1000 Genomes phase 3 reference panels specific to AFR, EAS (for ASN), EUR, AMR (for HIS) populations to compute LD for population-specific analyses. In MAGMA, the CPMA was conducted using the “all” 1000 Genomes phase 3 reference panel in the FUMA setting. For VEGAS2, we performed meta-analyses of population-specific gene-based results using Stouffer’s method, with p-values weighted by sample size. Gene-wide significance in MAGMA was defined as P < 2.61 × 10^− 6^, correcting for 19,122 protein-coding genes. VEGAS2 included 19,263 protein-coding genes, leading to a gene-wide significance threshold of P < 2.61 × 10^− 6^.

### Gene-set or Pathway-based analysis

We conducted gene-set analysis using MAGMA in FUMA to identify associations between gene sets and biological pathways. The analyses were performed based on the gene-based results from MAGMA, with statistical significance threshold at P < 2.94 × 10^− 6^, correcting for 17,009 gene sets. As a sensitivity analysis, we performed pathway-based analysis using VEGAS2Pathway [87], based on population-specific gene-based association results generated with VEGAS2. The meta-analyses were conducted using Stouffer’s method. VEGAS2Pathway included 2,748 pathways, resulting in a significance threshold of empirical P < 1.82 × 10^− 5^.

### Functional Annotations

All identified independent loci were assessed for potential functional annotations using multiple tools. First, we used the FUMA v1.5.2 to annotate functional information of the novel and known loci [84]. At the genomic region level, the FUMA SNP2GENE pipeline was used to prioritize genes based on the results of the top SNPs and SNPs in LD (r^2^ > 0.4 within 250 kb) through three gene mapping approaches: positional mapping, GTEx v8 eQTL mapping, and 3D chromatin interaction mapping (FDR ≤ 1 × 10^− 6^, 250bp upstream and 500bp downstream of the transcription start site [TSS] by default settings). At the variant level, we used QTLbase [88] and Open Target Genetics [89] databases to explore xQTL that link our loci to tissue or cell type specific functions. The xQTL include gene expression (eQTL), DNA methylation (mQTL), histone modification (hQTL), splicing event (sQTL), protein expression (pQTL), alternative polyadenylation (apaQTL), and others. To investigate whether the identified loci were associated with other phenotypes, we utilized a phenome-wide association studies (PheWAS) tool implemented in Open target genetics and GWAS ATLAS [90]. Using all the prioritized genes, we performed FUMA GENE2FUNC analysis to test enrichment of the gene sets and provide expression of those prioritized genes (adjusted p-value < 0.05).

### Druggability analyses

To assess the clinical potential of the candidate genes, we conducted integrative druggability analyses. We first used the Drug-Gene Interaction database (DGIdb; v4.2.0) to query high or medium priority and determine the potential druggability of the candidate gene targets. We annotated genes for implicated pathways and functions using the Kyoto Encyclopedia of Genes and Genomes (KEGG) database. We annotated the druggability target categories and queried all interacting drugs reported in 44 databases (Ensembl, HGNC, NCBI, ChemIDplus, Drugs@FDA, HemOnc, NCIt, RxNorm, Wikidata, CancerCommons, CGI, ChEMBL, CIViC, ClearityFoundationBiomarkers, ClearityFoundationClinicalTrial, COSMIC, DoCM, DrugBank, DTC, FDA, GuidetoPharmacology, JAX-CKB, MyCancerGenome, MyCancerGenomeClinicalTrial, OncoKB, PharmGKB, TALC, TdgClinicalTrial, TEND, TTD, BaderLab, CarisMolecularIntelligence, dGene, FoundationOneGenes, GO, HingoraniCasas, HopkinsGroom, HumanProteinAtlas, IDG, MskImpact, Oncomine, Pharos, RussLampel, Tempus). We queried protein targets for available active ligands in ChEMBL. We queried gene targets in the druggable genome using the most recent druggable genome list established from the NIH Illuminating the Druggable Genome Project (https://github.com/druggablegenome/IDGTargets) available through the Pharos web platform. We also queried FDA-approved drugs, late-stage clinical trials and disease indications in the DrugBank, ChEMBL, ClinicalTrials.gov databases and provided results for the top MESH and DrugBank indications and clinical trials.

## Figures and Tables

**Table 1:** Novel and known Loci associated with BP traits discovered through SNP × dDEPR interactions

Locus	CHR:position (hg38)	Alleles (E/A)	rsID	Analysis group	EAF	MAF AFR/EUR/ASN/HIS	Nearest gene	Position	Int Effect	Int SE	P Int	P Joint	P FDR^a^	P Het^b^	Sample size	P.Sex.Het
1q31.3	1:194548555	A/G	**rs1664073690** * ^▯^	CPMA-SBP	0.98	0/0.02/0/0.01	CDC73	intergenic	7.07	1.25	**1.44 × 10** ^**− 8**^	9.93 × 10^− 4^	0.09	0.30	30577	NA
2q13.3	2:70509396	C/T	**rs10178576** *	CPMA-PP	0.91	0.11/0/0/0.02	*TGFA*	intronic	2.59	0.46	**2.16 × 10** ^**− 8**^	5.11 × 10^− 7^	0.24	0.61	39482	0.56
3p22.1	3:42213248	G/T	rs115760284	CPMA-SBP	0.01	0.01/0/0/0.003	*TRAK1*	Intronic	−13.30	2.39	**2.78 × 10** ^**− 8**^	0.048	0.10	0.01	22241	NA
4q35.1	4:184777291	A/G	**rs113521945** *	CPMA-DBP	0.91	0.02/0.09/0.05/0.09	*ACSL1*	intronic	−0.57	0.10	**2.72 × 10** ^**− 8**^	5.74 × 10^− 7^	0.26	0.71	488129	0.05
7q21.11	7:78342531	A/T	rs147967138	ASN-PP	0.04	0/0/0.04/0	*MAGI2*	Intronic	5.12	0.93	**3.34 × 10** ^**− 8**^	2.89 × 10^− 7^	0.14	0.62	26307	0.8
7q31.1	7:112203372	A/G	rs757194	AFR-SBP	0.03	0.03/0/0/0.006	*DOCK4*	Intronic	13.62	2.58	1.39 **×** 10^− 7^	**7.99 × 10** ^**− 9**^	0.05	0.73	11644	NA
12p12.1	12:21435910	C/T	rs7979305	AFR-PP	0.95	0.05/0/0/0.007	*PYROXD1*	intergenic	−8.63	1.56	**3.09 × 10** ^**− 8**^	9.96 × 10^− 8^	0.18	0.26	13093	NA
13q32.1	13:96826633	A/G	rs75095906	CPMA-SBP	0.15	0.03/0.15/0.12/0.1	*HS6ST3*	Intronic	−0.76	0.14	**4.29 × 10** ^**− 8**^	2.45 × 10^− 5^	0.13	0.79	518557	0.99
16q23.2	16:81545886	C/T	rs9931605	CPMA-SBP	0.81	0.83/0.81/0.78/0.77	*CMIP*	Intronic	0.68	0.12	**1.36 × 10** ^**− 8**^	1.23 × 10^− 5^	0.09	0.86	543909	0.05

Allele E, effect allele; Allele A, non effect allele; EAF, effect allele frequency; MAF, minor allele frequency; AFR, African; EUR, European; ASN, Asian; HIS, Hispanic; Int Effect, interaction effects estimated in the 1df interaction test (Effect is in mmHg).

Int SE, standard error of interaction effects estimated in the 1df interaction test; P Int, P value of interaction effects in the 1df interaction test; P Joint, P value of joint effects of SNP main effect and interaction effect in 2df joint test; P.Sex.Het,

sex heterogeneity P value in two-sample Z tests

* **rs1664073690**, **rs10178576**, **rs113521945**: top SNPs at novel loci (at least 500 Kbp away from any previously reported BP locus)

▯ rs1664073690: absent in the 1000G Phase3 reference panels

a P.FDR: interaction FDR P value for 1df interaction test; joint FDR P value for 2df joint test

b P.Het: heterogeneity P value across population groups in CPMA; heterogeneity P value across studies in ancestry-specific meta-analyses

**Table 2:** Novel and known Loci associated with BP traits discovered through SNP × qDEPR interactions

Locus	CHR:position (hg38)	Alleles (E/A)	rsID	Analysis group	EAF	MAF AFR/EUR/ASN/HIS	Nearest gene	Position	Int Effect	Int SE	P Int	P Joint
2q14.2	2:118537183	A/G	rs77572777*	HIS-PP	0.99	0/0.02/0/0.01	*RP11–19E11.1*	intergenic	2.48	0.44	3.55 × 10^−5^	1.74 × 10^− 8^
3p26.3	3:1301059	C/T	rs148780833*^▯^	CPMA-SBP	0.01	0.01/0/0/0.002	*CNTN6*	intronic	5.94	1.04	9.91 × 10^−9^	2.85 × 10^− 9^
3p25.3	3:8726816	A/G	rs59284269	CPMA-SBP	0.09	0.23/0.02/0/0.06	*SSUH2*	intronic	0.86	0.16	4.29 × 10^−8^	8.65 × 10^− 7^
3q13.11	3:104214171	C/CA	rs748650739*^▯^	HIS-PP	0.99	0.05/0/0/0.01	*RP11–40M23.1*	intergenic	2.25	0.48	3.76 × 10^−5^	4.66 × 10^− 8^
4p14	4:39689605	C/T	rs145132348	AFR-DBP	0.02	0.03/0/0/0.006	*UBE2K*	intergenic	2.92	0.51	1.19 × 10^−8^	6.33 × 10^− 8^
12q13.13	12:52010638	C/T	rs114544309	CPMA-DBP	0.01	0.02/0/0/0.008	*GRASP*	intronic	2.45	0.44	1.85 × 10^−8^	2.56 × 10^− 7^
17p13.3	17:3225579	C/T	rs140618249*	CPMA-SBP	0.98	0.02/0/0/0.004	*OR1A1*	intergenic	−4.10	0.74	3.18 × 10^−8^	3.77 × 10^− 8^

Allele E, effect allele; Allele A, non effect allele; EAF, effect allele frequency; MAF, minor allele frequency; AFR, African; EUR, European; ASN, Asian; HIS, Hispanic; Int Effect, interaction effects estimated in the 1df interaction test (Effect is in mmHg).

Int SE, standard error of interaction effects estimated in the 1df interaction test; P Int, P value of interaction effects in the 1df interaction test; P Joint, P value of joint effects of SNP main effect and interaction effect in 2df joint test; P.Sex.Het,

sex heterogeneity P value in two-sample Z tests

* rs77572777, rs148780833, rs748650739, rs140618249: top SNPs at novel loci

▯ rs148780833, rs748650739: absent in the 1000G Phase3 reference panels

a P.FDR: Interaction FDR P value for 1df interaction test; Joint FDR P value for 2df joint test

b P.Het: Heterogeneity P value across population groups in CPMA; Heterogeneity P value across studies in ancestry-specific meta-analyses

## Data Availability

Due to restrictions in the written informed consent and local regulations, individual genotype-level data from this project could not be shared. Summary statistics are available at the CHARGE (Cohorts for Heart and Ageing Research in Genomics Epidemiology) dbGaP summary site (phs000930 [https://www.ncbi.nlm.nih.gov/projects/gap/cgi-bin/study.cgi?study_id=phs000930.v1.p1]).

## References

[1] Lawes CM, Vander Hoorn S, Rodgers A, International Society of H: Global burden of blood-pressure-related disease, 2001. Lancet 2008, 371:1513–1518. 18456100 10.1016/S0140-6736(08)60655-8

[2] Mills KT, Bundy JD, Kelly TN, Reed JE, Kearney PM, Reynolds K, Chen J, He J: Global Disparities of Hypertension Prevalence and Control: A Systematic Analysis of Population-Based Studies From 90 Countries. Circulation 2016, 134:441–450. 27502908 10.1161/CIRCULATIONAHA.115.018912 PMC4979614

[3] Jaeger BC, Chen L, Foti K, Hardy ST, Bress AP, Kane SP, Huang L, Herrick JS, Derington CG, Poudel B, : Hypertension Statistics for US Adults: An Open-Source Web Application for Analysis and Visualization of National Health and Nutrition Examination Survey Data. Hypertension 2023, 80:1311–1320. 37082970 10.1161/HYPERTENSIONAHA.123.20900 PMC10424908

[4] Aggarwal R, Chiu N, Wadhera RK, Moran AE, Raber I, Shen C, Yeh RW, Kazi DS: Racial/Ethnic Disparities in Hypertension Prevalence, Awareness, Treatment, and Control in the United States, 2013 to 2018. Hypertension 2021, 78:1719–1726. 34365809 10.1161/HYPERTENSIONAHA.121.17570 PMC10861176

[5] Hunter DJ: Gene-environment interactions in human diseases. Nat Rev Genet 2005, 6:287–298. 15803198 10.1038/nrg1578

[6] Manuck SB, McCaffery JM: Gene-environment interaction. Annu Rev Psychol 2014, 65:41–70. 24405358 10.1146/annurev-psych-010213-115100

[7] Virolainen SJ, VonHandorf A, Viel K, Weirauch MT, Kottyan LC: Gene-environment interactions and their impact on human health. Genes Immun 2023, 24:1–11. 36585519 10.1038/s41435-022-00192-6 PMC9801363

[8] Keaton JM, Kamali Z, Xie T, Vaez A, Williams A, Goleva SB, Ani A, Evangelou E, Hellwege JN, Yengo L, : Genome-wide analysis in over 1 million individuals of European ancestry yields improved polygenic risk scores for blood pressure traits. Nat Genet 2024, 56:778–791. 38689001 10.1038/s41588-024-01714-w PMC11096100

[9] McAllister K, Mechanic LE, Amos C, Aschard H, Blair IA, Chatterjee N, Conti D, Gauderman WJ, Hsu L, Hutter CM, : Current Challenges and New Opportunities for Gene-Environment Interaction Studies of Complex Diseases. Am J Epidemiol 2017, 186:753–761. 28978193 10.1093/aje/kwx227 PMC5860428

[10] Beilin LJ, Puddey IB, Burke V: Lifestyle and hypertension. Am J Hypertens 1999, 12:934–945. 10509554 10.1016/s0895-7061(99)00057-6

[11] Hardy ST, Sakhuja S, Jaeger BC, Oparil S, Akinyelure OP, Spruill TM, Kalinowski J, Butler M, Anstey DE, Elfassy T, : Maintaining Normal Blood Pressure Across the Life Course: The JHS. Hypertension 2021, 77:1490–1499. 33745299 10.1161/HYPERTENSIONAHA.120.16278 PMC8564773

[12] Nwanaji-Enwerem U, Onsomu EO, Roberts D, Singh A, Brummett BH, Williams RB, Dungan JR: Relationship Between Psychosocial Stress and Blood Pressure: The National Heart, Lung, and Blood Institute Family Heart Study. SAGE Open Nurs 2022, 8:23779608221107589. 10.1177/23779608221107589 PMC9234844 35769609

[13] Marwaha K: Examining the Role of Psychosocial Stressors in Hypertension. J Prev Med Public Health 2022, 55:499–505. 36475315 10.3961/jpmph.21.266 PMC9742403

[14] Meng L, Chen D, Yang Y, Zheng Y, Hui R: Depression increases the risk of hypertension incidence: a meta-analysis of prospective cohort studies. J Hypertens 2012, 30:842–851. 22343537 10.1097/HJH.0b013e32835080b7

[15] Schaare HL, Blochl M, Kumral D, Uhlig M, Lemcke L, Valk SL, Villringer A: Associations between mental health, blood pressure and the development of hypertension. Nat Commun 2023, 14:1953. 37029103 10.1038/s41467-023-37579-6 PMC10082210

[16] Xu Z, Wu X, Xiao C, Zhang W, Yan P, Yang C, Zhang L, Cui H, Tang M, Wang Y, : Observational and genetic analyses of the bidirectional relationship between depression and shypertension. J Affect Disord 2024, 348:62–69. 38123074 10.1016/j.jad.2023.12.028

[17] Sun D, Richard M, Musani SK, Sung YJ, Winkler TW, Schwander K, Chai JF, Guo X, Kilpelainen TO, Vojinovic D, : Multi-Ancestry Genome-wide Association Study Accounting for Gene-Psychosocial Factor Interactions Identifies Novel Loci for Blood Pressure Traits. HGG Adv 2021, 2. 10.1016/j.xhgg.2020.100013 PMC8562625 34734193

[18] Kavousi M, Bos MM, Barnes HJ, Lino Cardenas CL, Wong D, Lu H, Hodonsky CJ, Landsmeer LPL, Turner AW, Kho M, : Multi-ancestry genome-wide study identifies effector genes and druggable pathways for coronary artery calcification. Nat Genet 2023, 55:1651–1664. 37770635 10.1038/s41588-023-01518-4 PMC10601987

[19] Myles S, Davison D, Barrett J, Stoneking M, Timpson N: Worldwide population differentiation at disease-associated SNPs. BMC Med Genomics 2008, 1:22. 18533027 10.1186/1755-8794-1-22 PMC2440747

[20] Fei K, Rodriguez-Lopez JS, Ramos M, Islam N, Trinh-Shevrin C, Yi SS, Chernov C, Perlman SE, Thorpe LE: Racial and Ethnic Subgroup Disparities in Hypertension Prevalence, New York City Health and Nutrition Examination Survey, 2013–2014. Prev Chronic Dis 2017, 14:E33. 28427484 10.5888/pcd14.160478 PMC5420441

[21] Jones DW, Hall JE: Racial and ethnic differences in blood pressure: biology and sociology. Circulation 2006, 114:2757–2759. 17179032 10.1161/CIRCULATIONAHA.106.668731

[22] Ramsay M: Africa: continent of genome contrasts with implications for biomedical research and health. FEBS Lett 2012, 586:2813–2819. 22858376 10.1016/j.febslet.2012.07.061

[23] Singh S, Choudhury A, Hazelhurst S, Crowther NJ, Boua PR, Sorgho H, Agongo G, Nonterah EA, Micklesfield LK, Norris SA, : Genome-wide association study meta-analysis of blood pressure traits and hypertension in sub-Saharan African populations: an AWI-Gen study. Nat Commun 2023, 14:8376. 38104120 10.1038/s41467-023-44079-0 PMC10725455

[24] Takeuchi F, Akiyama M, Matoba N, Katsuya T, Nakatochi M, Tabara Y, Narita A, Saw WY, Moon S, Spracklen CN, : Interethnic analyses of blood pressure loci in populations of East Asian and European descent. Nat Commun 2018, 9:5052. 30487518 10.1038/s41467-018-07345-0 PMC6261994

[25] Sofer T, Wong Q, Hartwig FP, Taylor K, Warren HR, Evangelou E, Cabrera CP, Levy D, Kramer H, Lange LA, : Genome-Wide Association Study of Blood Pressure Traits by Hispanic/Latino Background: the Hispanic Community Health Study/Study of Latinos. Sci Rep 2017, 7:10348. 28871152 10.1038/s41598-017-09019-1 PMC5583292

[26] Licht CM, de Geus EJ, Seldenrijk A, van Hout HP, Zitman FG, van Dyck R, Penninx BW: Depression is associated with decreased blood pressure, but antidepressant use increases the risk for hypertension. Hypertension 2009, 53:631–638. 19237679 10.1161/HYPERTENSIONAHA.108.126698

[27] Shinn EH, Poston WS, Kimball KT, St Jeor ST, Foreyt JP: Blood pressure and symptoms of depression and anxiety: a prospective study. Am J Hypertens 2001, 14:660–664. 11482304 10.1016/s0895-7061(01)01304-8

[28] Krittanawong C, Maitra NS, Qadeer YK, Wang Z, Fogg S, Storch EA, Celano CM, Huffman JC, Jha M, Charney DS, Lavie CJ: Association of Depression and Cardiovascular Disease. Am J Med 2023, 136:881–895. 37247751 10.1016/j.amjmed.2023.04.036

[29] Spruill TM: Chronic psychosocial stress and hypertension. Curr Hypertens Rep 2010, 12:10–16. 20425153 10.1007/s11906-009-0084-8 PMC3694268

[30] Vyas CM, Donneyong M, Mischoulon D, Chang G, Gibson H, Cook NR, Manson JE, Reynolds CF 3rd, Okereke OI: Association of Race and Ethnicity With Late-Life Depression Severity, Symptom Burden, and Care. JAMA Netw Open 2020, 3:e201606. 32215634 10.1001/jamanetworkopen.2020.1606 PMC7325738

[31] Bailey RK, Mokonogho J, Kumar A: Racial and ethnic differences in depression: current perspectives. Neuropsychiatr Dis Treat 2019, 15:603–609. 30863081 10.2147/NDT.S128584 PMC6390869

[32] Fallon J, Reid S, Kinyamu R, Opole I, Opole R, Baratta J, Korc M, Endo TL, Duong A, Nguyen G, : In vivo induction of massive proliferation, directed migration, and differentiation of neural cells in the adult mammalian brain. Proc Natl Acad Sci U S A 2000, 97:14686–14691. 11121069 10.1073/pnas.97.26.14686 PMC18979

[33] Lazar LM, Blum M: Regional distribution and developmental expression of epidermal growth factor and transforming growth factor-alpha mRNA in mouse brain by a quantitative nuclease protection assay. J Neurosci 1992, 12:1688–1697. 1578263 10.1523/JNEUROSCI.12-05-01688.1992 PMC6575894

[34] Bialek K, Czarny P, Watala C, Wigner P, Talarowska M, Galecki P, Szemraj J, Sliwinski T: Novel association between TGFA, TGFB1, IRF1, PTGS2 and IKBKB single-nucleotide polymorphisms and occurrence, severity and treatment response of major depressive disorder. PeerJ 2020, 8:e8676. 32140313 10.7717/peerj.8676 PMC7047865

[35] Dai X, Chen J, Xu F, Zhao J, Cai W, Sun Z, Hitchens TK, Foley LM, Leak RK, Chen J, Hu X: TGFalpha preserves oligodendrocyte lineage cells and improves white matter integrity after cerebral ischemia. J Cereb Blood Flow Metab 2020, 40:639–655. 30834805 10.1177/0271678X19830791 PMC7026842

[36] Bialek K, Czarny P, Wigner P, Synowiec E, Barszczewska G, Bijak M, Szemraj J, Niemczyk M, Tota-Glowczyk K, Papp M, Sliwinski T: Chronic Mild Stress and Venlafaxine Treatment Were Associated with Altered Expression Level and Methylation Status of New Candidate Inflammatory Genes in PBMCs and Brain Structures of Wistar Rats. Genes (Basel) 2021, 12. 10.3390/genes12050667 PMC8146372 33946816

[37] Li QS, Wajs E, Ochs-Ross R, Singh J, Drevets WC: Genome-wide association study and polygenic risk score analysis of esketamine treatment response. Sci Rep 2020, 10:12649. 32724131 10.1038/s41598-020-69291-6 PMC7387452

[38] Wang CH, Surbhi, Goraya S, Byun J, Pennathur S: Fatty acids and inflammatory stimuli induce expression of long-chain acyl-CoA synthetase 1 to promote lipid remodeling in diabetic kidney disease. J Biol Chem 2024, 300:105502. 38016515 10.1016/j.jbc.2023.105502 PMC10770716

[39] Chen Y, He L, Yang Y, Chen Y, Song Y, Lu X, Liang Y: The inhibition of Nrf2 accelerates renal lipid deposition through suppressing the ACSL1 expression in obesity-related nephropathy. Ren Fail 2019, 41:821–831. 31488013 10.1080/0886022X.2019.1655450 PMC6735294

[40] Wadei HM, Textor SC: The role of the kidney in regulating arterial blood pressure. Nat Rev Nephrol 2012, 8:602–609. 22926246 10.1038/nrneph.2012.191

[41] Tzeng TT, Tsay HJ, Chang L, Hsu CL, Lai TH, Huang FL, Shiao YJ: Caspase 3 involves in neuroplasticity, microglial activation and neurogenesis in the mice hippocampus after intracerebral injection of kainic acid. J Biomed Sci 2013, 20:90. 24313976 10.1186/1423-0127-20-90 PMC4028745

[42] Toledano A, Alvarez MI, Caballero I, Carmona P, De Miguel E: Immunohistochemical increase in cyclooxygenase-2 without apoptosis in different brain areas of subchronic nicotine- and D-amphetamine-treated rats. J Neural Transm (Vienna) 2008, 115:1093–1108. 18351285 10.1007/s00702-008-0040-9

[43] D’Amelio M, Cavallucci V, Cecconi F: Neuronal caspase-3 signaling: not only cell death. Cell Death Differ 2010, 17:1104–1114. 19960023 10.1038/cdd.2009.180

[44] Gervais FG, Xu D, Robertson GS, Vaillancourt JP, Zhu Y, Huang J, LeBlanc A, Smith D, Rigby M, Shearman MS, : Involvement of caspases in proteolytic cleavage of Alzheimer’s amyloid-beta precursor protein and amyloidogenic A beta peptide formation. Cell 1999, 97:395–406. 10319819 10.1016/s0092-8674(00)80748-5

[45] Blizniewska-Kowalska K, Galecki P, Szemraj J, Su KP, Chang JP, Galecka M: CASP3 gene expression and the role of caspase 3 in the pathogenesis of depressive disorders. BMC Psychiatry 2023, 23:656. 37674109 10.1186/s12888-023-05153-5 PMC10481541

[46] Zuko A, Oguro-Ando A, van Dijk R, Gregorio-Jordan S, van der Zwaag B, Burbach JP: Developmental role of the cell adhesion molecule Contactin-6 in the cerebral cortex and hippocampus. Cell Adh Migr 2016, 10:378–392. 26939565 10.1080/19336918.2016.1155018 PMC4986711

[47] Mercati O, Huguet G, Danckaert A, Andre-Leroux G, Maruani A, Bellinzoni M, Rolland T, Gouder L, Mathieu A, Buratti J, : CNTN6 mutations are risk factors for abnormal auditory sensory perception in autism spectrum disorders. Mol Psychiatry 2017, 22:625–633. 27166760 10.1038/mp.2016.61 PMC5378808

[48] Everlien I, Yen TY, Liu YC, Di Marco B, Vazquez-Marin J, Centanin L, Alfonso J, Monyer H: Diazepam binding inhibitor governs neurogenesis of excitatory and inhibitory neurons during embryonic development via GABA signaling. Neuron 2022, 110:3139–3153 e3136. 35998632 10.1016/j.neuron.2022.07.022

[49] Dong E, Matsumoto K, Tohda M, Kaneko Y, Watanabe H: Diazepam binding inhibitor (DBI) gene expression in the brains of socially isolated and group-housed mice. Neurosci Res 1999, 33:171–177. 10211760 10.1016/s0168-0102(99)00010-3

[50] Montegut L, Joseph A, Chen H, Abdellatif M, Ruckenstuhl C, Motino O, Lambertucci F, Anagnostopoulos G, Lachkar S, Dichtinger S, : High plasma concentrations of acyl-coenzyme A binding protein (ACBP) predispose to cardiovascular disease: Evidence for a phylogenetically conserved proaging function of ACBP. Aging Cell 2023, 22:e13751. 36510662 10.1111/acel.13751 PMC9835587

[51] Hiramoto-Yamaki N, Takeuchi S, Ueda S, Harada K, Fujimoto S, Negishi M, Katoh H: Ephexin4 and EphA2 mediate cell migration through a RhoG-dependent mechanism. J Cell Biol 2010, 190:461–477. 20679435 10.1083/jcb.201005141 PMC2922637

[52] Guo D, Peng Y, Wang L, Sun X, Wang X, Liang C, Yang X, Li S, Xu J, Ye WC, : Autism-like social deficit generated by Dock4 deficiency is rescued by restoration of Rac1 activity and NMDA receptor function. Mol Psychiatry 2021, 26:1505–1519. 31388105 10.1038/s41380-019-0472-7 PMC8159750

[53] Autism Spectrum Disorders Working Group of The Psychiatric Genomics C: Meta-analysis of GWAS of over 16,000 individuals with autism spectrum disorder highlights a novel locus at 10q24.32 and a significant overlap with schizophrenia. Mol Autism 2017, 8:21. 28540026 10.1186/s13229-017-0137-9 PMC5441062

[54] Athanasiu L, Smorr LL, Tesli M, Rossberg JI, Sonderby IE, Spigset O, Djurovic S, Andreassen OA: Genome-wide association study identifies common variants associated with pharmacokinetics of psychotropic drugs. J Psychopharmacol 2015, 29:884–891. 25944848 10.1177/0269881115584469

[55] Almuwaqqat Z, Liu C, Kim JH, Hammadah M, Alkhoder A, Raggi P, Shah AJ, Bremner JD, Vaccarino V, Sun YV, Quyyumi AA: A novel GWAS locus influences microvascular response to mental stress and predicts adverse cardiovascular events. Sci Rep 2024, 14:23479. 39379420 10.1038/s41598-024-54566-z PMC11461842

[56] Goes FS, McGrath J, Avramopoulos D, Wolyniec P, Pirooznia M, Ruczinski I, Nestadt G, Kenny EE, Vacic V, Peters I, : Genome-wide association study of schizophrenia in Ashkenazi Jews. Am J Med Genet B Neuropsychiatr Genet 2015, 168:649–659. 26198764 10.1002/ajmg.b.32349

[57] Meng X, Navoly G, Giannakopoulou O, Levey DF, Koller D, Pathak GA, Koen N, Lin K, Adams MJ, Renteria ME, : Multi-ancestry genome-wide association study of major depression aids locus discovery, fine mapping, gene prioritization and causal inference. Nat Genet 2024, 56:222–233. 38177345 10.1038/s41588-023-01596-4 PMC10864182

[58] Divers J, Palmer ND, Langefeld CD, Brown WM, Lu L, Hicks PJ, Smith SC, Xu J, Terry JG, Register TC, : Genome-wide association study of coronary artery calcified atherosclerotic plaque in African Americans with type 2 diabetes. BMC Genet 2017, 18:105. 29221444 10.1186/s12863-017-0572-9 PMC5723099

[59] Hirao K, Hata Y, Ide N, Takeuchi M, Irie M, Yao I, Deguchi M, Toyoda A, Sudhof TC, Takai Y: A novel multiple PDZ domain-containing molecule interacting with N-methyl-D-aspartate receptors and neuronal cell adhesion proteins. J Biol Chem 1998, 273:21105–21110. 9694864 10.1074/jbc.273.33.21105

[60] Huang SS, Chen YT, Su MH, Tsai SJ, Chen HH, Yang AC, Liu YL, Kuo PH: Investigating genetic variants for treatment response to selective serotonin reuptake inhibitors in syndromal factors and side effects among patients with depression in Taiwanese Han population. Pharmacogenomics J 2023, 23:50–59. 36658263 10.1038/s41397-023-00298-8

[61] Pinakhina D, Yermakovich D, Vergasova E, Kasyanov E, Rukavishnikov G, Rezapova V, Kolosov N, Sergushichev A, Popov I, Kovalenko E, : GWAS of depression in 4,520 individuals from the Russian population highlights the role of MAGI2 (S-SCAM) in the gut-brain axis. Front Genet 2022, 13:972196. 36685848 10.3389/fgene.2022.972196 PMC9845291

[62] Coleman JRI, Peyrot WJ, Purves KL, Davis KAS, Rayner C, Choi SW, Hubel C, Gaspar HA, Kan C, Van der Auwera S, : Genome-wide gene-environment analyses of major depressive disorder and reported lifetime traumatic experiences in UK Biobank. Mol Psychiatry 2020, 25:1430–1446. 31969693 10.1038/s41380-019-0546-6 PMC7305950

[63] Hodgkinson CA, Enoch MA, Srivastava V, Cummins-Oman JS, Ferrier C, Iarikova P, Sankararaman S, Yamini G, Yuan Q, Zhou Z, : Genome-wide association identifies candidate genes that influence the human electroencephalogram. Proc Natl Acad Sci U S A 2010, 107:8695–8700. 20421487 10.1073/pnas.0908134107 PMC2889314

[64] Del Boca C, Lutz PE, Le Merrer J, Koebel P, Kieffer BL: Cholecystokinin knock-down in the basolateral amygdala has anxiolytic and antidepressant-like effects in mice. Neuroscience 2012, 218:185–195. 22613736 10.1016/j.neuroscience.2012.05.022 PMC3532740

[65] Becker C, Zeau B, Rivat C, Blugeot A, Hamon M, Benoliel JJ: Repeated social defeat-induced depression-like behavioral and biological alterations in rats: involvement of cholecystokinin. Mol Psychiatry 2008, 13:1079–1092. 17893702 10.1038/sj.mp.4002097

[66] Lofberg C, Agren H, Harro J, Oreland L: Cholecystokinin in CSF from depressed patients: possible relations to severity of depression and suicidal behaviour. Eur Neuropsychopharmacol 1998, 8:153–157. 9619694 10.1016/s0924-977x(97)00046-1

[67] Goetze JP, Rehfeld JF, Alehagen U: Cholecystokinin in plasma predicts cardiovascular mortality in elderly females. Int J Cardiol 2016, 209:37–41. 26878472 10.1016/j.ijcard.2016.02.038

[68] Koyama S, Fujita T, Shibamoto T, Matsuda Y, Uematsu H, Jones RO: Contribution of baroreceptor reflexes to blood pressure and sympathetic responses to cholecystokinin and vasoactive intestinal peptide in anesthetized dogs. Eur J Pharmacol 1990, 175:245–251. 2323348 10.1016/0014-2999(90)90561-j

[69] Hagenbuch B, Gao B, Meier PJ: Transport of xenobiotics across the blood-brain barrier. News Physiol Sci 2002, 17:231–234. 12433976 10.1152/nips.01402.2002

[70] Gao B, Hagenbuch B, Kullak-Ublick GA, Benke D, Aguzzi A, Meier PJ: Organic anion-transporting polypeptides mediate transport of opioid peptides across blood-brain barrier. J Pharmacol Exp Ther 2000, 294:73–79. 10871297

[71] Schafer AM, Meyer Zu Schwabedissen HE, Grube M: Expression and Function of Organic Anion Transporting Polypeptides in the Human Brain: Physiological and Pharmacological Implications. Pharmaceutics 2021, 13. 10.3390/pharmaceutics13060834 PMC8226904 34199715

[72] Sanchez-Roige S, Fontanillas P, Elson SL, Gray JC, de Wit H, MacKillop J, Palmer AA: Genome-Wide Association Studies of Impulsive Personality Traits (BIS-11 and UPPS-P) and Drug Experimentation in up to 22,861 Adult Research Participants Identify Loci in the CACNA1I and CADM2 genes. J Neurosci 2019, 39:2562–2572. 30718321 10.1523/JNEUROSCI.2662-18.2019 PMC6435820

[73] Karlsson Linner R, Biroli P, Kong E, Meddens SFW, Wedow R, Fontana MA, Lebreton M, Tino SP, Abdellaoui A, Hammerschlag AR, : Genome-wide association analyses of risk tolerance and risky behaviors in over 1 million individuals identify hundreds of loci and shared genetic influences. Nat Genet 2019, 51:245–257. 30643258 10.1038/s41588-018-0309-3 PMC6713272

[74] Rao DC, Sung YJ, Winkler TW, Schwander K, Borecki I, Cupples LA, Gauderman WJ, Rice K, Munroe PB, Psaty BM, Group* CG-LIW: Multiancestry Study of Gene-Lifestyle Interactions for Cardiovascular Traits in 610 475 Individuals From 124 Cohorts: Design and Rationale. Circ Cardiovasc Genet 2017, 10. 10.1161/CIRCGENETICS.116.001649 PMC5476223 28620071

[75] Constantinescu AE, Mitchell RE, Zheng J, Bull CJ, Timpson NJ, Amulic B, Vincent EE, Hughes DA: A framework for research into continental ancestry groups of the UK Biobank. Hum Genomics 2022, 16:3. 35093177 10.1186/s40246-022-00380-5 PMC8800339

[76] Newton-Cheh C, Johnson T, Gateva V, Tobin MD, Bochud M, Coin L, Najjar SS, Zhao JH, Heath SC, Eyheramendy S, : Genome-wide association study identifies eight loci associated with blood pressure. Nat Genet 2009, 41:666–676. 19430483 10.1038/ng.361 PMC2891673

[77] Tobin MD, Sheehan NA, Scurrah KJ, Burton PR: Adjusting for treatment effects in studies of quantitative traits: antihypertensive therapy and systolic blood pressure. Stat Med 2005, 24:2911–2935. 16152135 10.1002/sim.2165

[78] Keller MC: Gene × environment interaction studies have not properly controlled for potential confounders: the problem and the (simple) solution. Biol Psychiatry 2014, 75:18–24. 24135711 10.1016/j.biopsych.2013.09.006 PMC3859520

[79] Kraft P, Yen YC, Stram DO, Morrison J, Gauderman WJ: Exploiting gene-environment interaction to detect genetic associations. Hum Hered 2007, 63:111–119. 17283440 10.1159/000099183

[80] Willer CJ, Li Y, Abecasis GR: METAL: fast and efficient meta-analysis of genomewide association scans. Bioinformatics 2010, 26:2190–2191. 20616382 10.1093/bioinformatics/btq340 PMC2922887

[81] Manning AK, LaValley M, Liu CT, Rice K, An P, Liu Y, Miljkovic I, Rasmussen-Torvik L, Harris TB, Province MA, : Meta-analysis of gene-environment interaction: joint estimation of SNP and SNP × environment regression coefficients. Genet Epidemiol 2011, 35:11–18. 21181894 10.1002/gepi.20546 PMC3312394

[82] Winkler TW, Kutalik Z, Gorski M, Lottaz C, Kronenberg F, Heid IM: EasyStrata: evaluation and visualization of stratified genome-wide association meta-analysis data. Bioinformatics 2015, 31:259–261. 25260699 10.1093/bioinformatics/btu621 PMC4287944

[83] Altman DG, Bland JM: Interaction revisited: the difference between two estimates. BMJ 2003, 326:219. 12543843 10.1136/bmj.326.7382.219 PMC1125071

[84] Watanabe K, Taskesen E, van Bochoven A, Posthuma D: Functional mapping and annotation of genetic associations with FUMA. Nat Commun 2017, 8:1826. 29184056 10.1038/s41467-017-01261-5 PMC5705698

[85] Mishra A, Macgregor S: VEGAS2: Software for More Flexible Gene-Based Testing. Twin Res Hum Genet 2015, 18:86–91. 25518859 10.1017/thg.2014.79

[86] de Leeuw CA, Mooij JM, Heskes T, Posthuma D: MAGMA: generalized gene-set analysis of GWAS data. PLoS Comput Biol 2015, 11:e1004219. 25885710 10.1371/journal.pcbi.1004219 PMC4401657

[87] Mishra A, MacGregor S: A Novel Approach for Pathway Analysis of GWAS Data Highlights Role of BMP Signaling and Muscle Cell Differentiation in Colorectal Cancer Susceptibility. Twin Res Hum Genet 2017, 20:1–9. 28105966 10.1017/thg.2016.100

[88] Huang D, Feng X, Yang H, Wang J, Zhang W, Fan X, Dong X, Chen K, Yu Y, Ma X, : QTLbase2: an enhanced catalog of human quantitative trait loci on extensive molecular phenotypes. Nucleic Acids Res 2023, 51:D1122–D1128. 36330927 10.1093/nar/gkac1020 PMC9825467

[89] Ghoussaini M, Mountjoy E, Carmona M, Peat G, Schmidt EM, Hercules A, Fumis L, Miranda A, Carvalho-Silva D, Buniello A, : Open Targets Genetics: systematic identification of trait-associated genes using large-scale genetics and functional genomics. Nucleic Acids Res 2021, 49:D1311–D1320. 33045747 10.1093/nar/gkaa840 PMC7778936

[90] Watanabe K, Stringer S, Frei O, Umicevic Mirkov M, de Leeuw C, Polderman TJC, van der Sluis S, Andreassen OA, Neale BM, Posthuma D: A global overview of pleiotropy and genetic architecture in complex traits. Nat Genet 2019, 51:1339–1348. 31427789 10.1038/s41588-019-0481-0

